# Editorial: Antibody-Mediated Autoimmune Diseases of the CNS: Challenges and Approaches to Diagnosis and Management

**DOI:** 10.3389/fneur.2022.844155

**Published:** 2022-03-02

**Authors:** Yin-Xi Zhang, Wei Qiu, Hong-Zhi Guan, Long-Jun Wu, Mei-Ping Ding

**Affiliations:** ^1^Department of Neurology, Second Affiliated Hospital, School of Medicine, Zhejiang University, Hangzhou, China; ^2^Department of Neurology, Third Affiliated Hospital of Sun Yat-sen University, Guangzhou, China; ^3^Department of Neurology, Peking Union Medical College Hospital, Chinese Academy of Medical Sciences and Peking Union Medical College, Beijing, China; ^4^Department of Neurology, Mayo Clinic, Rochester, MN, United States

**Keywords:** antibody, autoimmune encephalitis, demyelinating disease, central nervous system, neuroimmunology

Antibody-mediated central nervous system (CNS) autoimmune diseases are an important part of neuroimmunology research, and mainly affect young people, are notoriously complex, and have a high risk of disability. With recent advancements in detection technology, many autoantibodies targeting neurons or glia have been identified, providing the possibility for precise etiological diagnoses. As a result, the spectrum of antibody-mediated CNS diseases, ranging from CNS demyelination to autoimmune encephalitis is expanding. This brings with it a series of interesting research topics to investigate the immunopathogenesis of these diseases. As different CNS autoantibodies have distinct pathophysiologic mechanisms, patients may present a variety of manifestations, posing a challenge to diagnosis, and management. Therefore, accurate detection, objective evaluation, and identification of CNS autoantibodies is key to proper diagnosis and the development of effective treatments. To provide a platform for sharing the latest clinical progress and research findings in the field of antibody-mediated CNS autoimmune diseases, we organized this special issue, in which 39 manuscripts have been accepted for publication, including 21 original research articles, 2 brief research reports, 3 reviews, 2 mini reviews, 1 meta-analysis and systematic review, and 10 case reports. These studies have greatly expanded our current understanding of antibody-mediated autoimmune diseases of the CNS.

Neuromyelitis optica spectrum disorder (NMOSD) is a key representative of antibody-mediated CNS demyelinating diseases and preferentially affects the optic nerves and spinal cord. Serum aquaporin 4 (AQP4) antibodies can be detected in most patients with NMOSD (1). Common manifestations include relapsing episodes of transverse myelitis and optic neuritis, which leads to severe disabilities such as paralysis and blindness. High-dose corticosteroids are the first-line therapy for acute attacks in NMOSD (2). However, a proportion of patients respond poorly to this form of intervention. Qin et al. discovered that previous use of immune-suppressants, higher levels of cerebrospinal fluid (CSF) proteins, and active lesions in the brainstem were all predictors of corticosteroid resistance. For these patients, it is very important to initiate other effective treatment as early as possible. Oral corticosteroid maintenance is usually required to prevent relapse after intervention during the acute period. A study by Akaishi et al. focused on the impact of oral corticosteroid therapy in NMOSD patients with symptoms including depression and chronic fatigue. Their results suggested that the use of long-term low- to medium-dose oral prednisolone did not aggravate the psychiatric and fatigue conditions. Instead, early initiation of oral prednisolone might alleviate the subsequent depressive and fatigue conditions due to this disease. Since there are currently limited treatment protocols for NMOSD, researchers tested other therapeutic interventions to improve prognosis. Wang, Liu, Tan, et al. retrospectively explored remedial treatments in corticosteroid-refractory NMOSD patients during the acute phase of optic neuritis (ON). The authors concluded that cyclophosphamide might be safe and effective within 30 days of attack onset, which is good news to NMOSD-ON patients resistant to corticosteroid therapy. In addition, Du et al. conducted the first retrospective study to investigate the effects of tocilizumab, a monoclonal antibody that inhibits interleukin-6 signaling, during both acute attacks and the maintenance phase of NMOSD patients with moderate-to-severe myelitis. They found that early initiation of tocilizumab provided a safe and effective add-on therapy during attacks and can be followed by regular infusions. This strategy may provide new insights into treatment options for NMOSD. In Xue et al.'s meta-analysis of randomized controlled trials, monoclonal antibody therapies including tocilizumab, satralizumab, rituximab, inebilizumab, and eculizumab were analyzed, and were found effective and safe, further supporting the possible use of these antibody therapies in NMOSD.

Over the past few years, antibodies directed against myelin oligodendrocyte glycoprotein (MOG) have been detected in CNS demyelinating disorders. MOG antibody-associated disease (MOGAD), which was recently recognized as a distinct nosological entity, covers a wide spectrum of clinical manifestations and radiological patterns (3). Zheng et al. reported a case with recurrent episodes of short-segment myelitis typical of multiple sclerosis (MS), but was later diagnosed as MOGAD. This case highlights the potential overlap of clinical presentations in MOGAD and MS, and MOG antibody testing in all patients with recurrent short-segment myelitis, conus medullaris involvement, and those who demonstrated corticosteroid dependence is recommended. Wang, Liu, Lin, et al. presented 2 new cases of the rare phenotype of MOGAD, named fluid attenuated inversion recovery-hyperintense lesions in anti-MOG antibody-associated cerebral cortical encephalitis with seizures and conducted a review of previously described cases to further characterize this distinct clinico-radiographic syndrome. Fujimori et al. reviewed another rare bilateral medial frontal cortical encephalitis with anti-MOG antibodies, with characteristic clinical symptoms, such as paraparesis, lethargy, and memory decline, and radiological findings, such as lesion formations in the anterior cerebral artery territory. These articles expand our recognition and understanding of these rare phenotypes of MOGAD. Moreover, Gao et al. evaluated structural and functional alterations in the visual pathway after ON attack in MOGAD, and compared those with NMOSD-ON. This prospective study provided evidence that differed from the imaging manifestations of AQP4-ON. The structural integrity of optic radiation in MOG-ON might be a reason for the better visual outcomes in these patients.

Accurate detection of MOG and AQP4 antibodies is crucial for the diagnosis of MOGAD and NMOSD. Live cell-based assays (CBA) performed via indirect immunofluorescence microscopy (IF) or flow cytometry (FC) are currently considered the “gold standard” (4–6). Marchionatti et al. compared the performance of CBA-IF and CBA-FC analysis in the detection of MOG antibodies. Both techniques can be used in clinical practice, each with different advantages and limitations. The combination of both CBA-IF and CBA-FC could improve detection accuracy. In addition, Bollo et al. reported that semiquantitative ratiometric methods could accurately detect even slight variation of MOG and AQP4 antibody titers during the disease course.

Autoimmune encephalitis (AE) is a group of non-infectious, immune-mediated brain parenchymal inflammatory diseases. These diseases are characterized by a wide spectrum of clinical manifestations associated with different neural antibodies, including seizures, loss of memory, psychosis, changes in behavior, and impaired consciousness. In the past 10 years, research into AE, especially neuronal surface antibody-mediated AE, has made a significant progress. However, multi-center studies with larger sample sizes are still lacking. Shan et al. reviewed information collected from 778 patients with neuronal surface antibody-mediated AE from across 22 centers in China. The retrospective study provided a detailed description of demographic and clinical characteristics, immunotherapy regimens, and long-term outcomes of multiple subtypes of AE. Qiao et al. performed a retrospective analysis of the epidemiological characteristics, clinical manifestations, treatments, outcomes, and prognostic factors of 185 AE patients with neuronal surface antibodies from 5 clinical centers in the east of China. Separately, Swayne et al. presented the findings from Australia analyzing AE cases, in which clinical data, therapeutic approach, treatment response, and outcome measures across a range of AE subsets were assessed and compared. Hayden et al. conducted a retrospective study within a multi-center cohort of Hungarian patients to characterize the clinical features and outcomes of neuronal surface antibody-mediated AE. Moreover, Sun et al. (a) demonstrated that age, viral prodrome, consciousness impairment, memory dysfunction, and autonomic dysfunction were significant predictors for poor prognosis of AE at discharge based on a multi-center retrospective study of 173 patients from 4 hospitals in China. Additionally, they developed and validated a reliable nomogram for predicting prognosis, which might help provide an early and accurate prediction of prognosis in patients with AE. A prospective observational study by Seifert-Held et al. focused on functional recovery in AE, demonstrating the different clinical dynamics of AE. All of these studies help to improve our understanding of AE and its subtypes and enhance clinical diagnosis and management.

Anti-N-methyl-D-aspartate receptor (NMDAR) encephalitis is the most common type of AE (7). Liu et al. analyzed the results from urinalysis and renal function between 82 patients with anti-NMDAR encephalitis and 166 age- and sex-matched healthy controls for the first time. The study showed that urine pH levels were higher in anti-NMDAR encephalitis patients while urine specific gravity levels were lower in these patients when compared with healthy controls. These findings indicated that the pathophysiological changes related to anti-NMDAR encephalitis are not limited to the CNS. Another study by Sun et al. (b) evaluated the association between age and prognosis in anti-NMDAR encephalitis patients under the age of 45. They concluded that increasing age was associated with a poorer prognosis in female patients. In a study focusing on pediatric anti-NMDAR encephalitis, Dou et al. retrospectively recruited 8 Chinese children with refractory anti-NMDAR encephalitis and treated with rituximab as a second-line immunotherapy. The study revealed that rituximab could improve the clinical outcomes, suggesting it is a potential treatment option for these patients.

Anti-leucine-rich glioma-inactivated 1 (LGI1) encephalitis is the second most common form of AE. The disease is also the most common autoimmune limbic encephalitis, with faciobrachial dystonic seizure (FBDS) as one of the most specific symptoms and abnormal signals often seen on neuroimaging (7). Shao et al. while correlating brain magnetic resonance imaging (MRI) features to clinical relevance found the basal ganglia was an important structure affected by anti-LGI1 encephalitis in addition to medial temporal lobe, having implications in FBDS. The study suggested that MRI was not only helpful for diagnosis, but also a useful tool to quantify disease severity. Another imaging study by Zhao et al. explored the glucose metabolic patterns of this disorder based on positron emission tomography voxel analysis. They found that hippocampal and basal ganglia hypermetabolism co-existed with neocortical hypometabolism and was a common metabolic abnormality in anti-LGI1 encephalitis. Furthermore, the extent of the metabolic gradient between the hippocampus and neocortical regions was positively correlated with neurological disability and that basal ganglia hypermetabolism might contribute to the development of FBDS.

Additionally, Qin et al. retrospectively examined 25 patients with confirmed anti-contactin-associated protein-2 (CASPR2) antibody-associated AE from 5 medical centers in China to identify clinical and paraclinical characteristics. Zhang et al. reviewed and summarized the clinical presentations, diagnostic results, and treatments of anti-alpha-amino-3-hydroxy-5-methyl-4-isoxazolepropionic acid receptor encephalitis. Ren, Ren, et al.  reported the first case series of AE associated with anti-glutamic acid decarboxylase antibodies in Chinese children. Although these antibody-mediated AE are relatively rare, they should still be recognized in a clinical setting.

Coexistence with autoimmune syndromes and/or multiple autoantibodies has been observed in AE patients. Li et al. reported a patient with a 15-year history of primary Sjögren's syndrome presenting with progressive cognitive dysfunction attributable to anti-NMDAR encephalitis, emphasizing the need for AE awareness in this condition. Huang et al. described a patient diagnosed with relapsing remitting MS who developed anti-NMDAR encephalitis. Separately, Zhou et al. presented a case of a young female who was initially diagnosed with anti-NMDAR encephalitis, but was later re-diagnosed as MS during a second relapse. These observations suggested that there might be a possible link between anti-NMDAR encephalitis and MS. Ren, Guo, et al.  reported a patient diagnosed with anti-NMDAR encephalitis combined with MOGAD. This case implied that comprehensive autoantibody analysis should be conducted on patients with CNS demyelination relapse. In addition, Otiniano-Sifuentes et al. described a patient with anti-LGI1 encephalitis with concurrent anti-thyroid antibodies, which can also be detected in other types of AE.

Immune checkpoint inhibitor (ICI) therapy is an emerging antineoplastic treatment approach that has shown powerful effects and broad application prospects for the treatment of malignant tumors. With the introduction and promotion of ICI cancer immunotherapy in the clinic, there has been an increase in autoimmune neurological events in patients with malignancies not traditionally associated with paraneoplastic neurological syndromes. Valencia-Sanchez et al. reviewed the pathogenic mechanisms, clinical presentation, diagnostic approach, and therapeutic management of these complications. Clinicians must be aware of the diverse spectrum of neurological toxicities associated with ICI therapy, for instance, AE and CNS demyelination. Early recognition and treatment is critical to avoid worsening outcomes.

Finally, the comprehensive review of antibody-mediated autoimmune diseases of the CNS by Sechi et al. summarized the main clinical syndromes and MRI characteristics associated with neural antibodies, and discussed the challenges to diagnosis and management. They concluded that these disorders have variable clinical-imaging characteristics based on the specific underlying neural autoantibody. One or more neural antibodies detected in the serum or CSF of patients with a compatible clinical phenotype can confirm the diagnosis, but the possibility of false positives and false negatives should be considered. Current treatment strategies for antibody-mediated CNS disorders are often broad immunotherapy. Future studies with an emphasis on targeted treatments are still needed.

In summary, this collection of articles provides novel scientific evidence that advance our understanding of clinical presentation, neuroimaging, antibody analysis, and disease mechanisms of antibody-mediated autoimmune CNS diseases. Moreover, these papers highlight enormous recent progress of diagnostic approaches and therapeutic regimens, as well as the remaining challenges.

## Author Contributions

All authors listed have made a substantial, direct, and intellectual contribution to the work and approved it for publication.

## Conflict of Interest

The authors declare that the research was conducted in the absence of any commercial or financial relationships that could be construed as a potential conflict of interest.

## Publisher's Note

All claims expressed in this article are solely those of the authors and do not necessarily represent those of their affiliated organizations, or those of the publisher, the editors and the reviewers. Any product that may be evaluated in this article, or claim that may be made by its manufacturer, is not guaranteed or endorsed by the publisher.

## References

[1] Wingerchuk DM Banwell B Bennett JL Cabre P Carroll W Chitnis T. International consensus diagnostic criteria for neuromyelitis optica spectrum disorders. Neurology. (2015) 85:177–89. 10.1212/WNL.0000000000001729 26092914 PMC4515040

[2] Jarius S Paul F Weinshenker BG Levy M Kim HJ Wildemann B. Neuromyelitis optica. Nat Rev Dis Primers. (2020) 6:85. 10.1038/s41572-020-0214-9 33093467

[3] Lai QL Zhang YX Cai MT Zheng Y Qiao S Fang GL. Efficacy and safety of immunosuppressive therapy in myelin oligodendrocyte glycoprotein antibody-associated disease: a systematic review and meta-analysis. Ther Adv Neurol Disord. (2021) 14:17562864211054157. 10.1177/17562864211054157 34790259 PMC8591780

[4] Waters P Reindl M Saiz A Schanda K Tuller F Kral V. Multicentre comparison of a diagnostic assay: aquaporin-4 antibodies in neuromyelitis optica. J Neurol Neurosurg Psychiatry. (2016) 87:1005–15. 10.1136/jnnp-2015-312601 27113605 PMC5013123

[5] Reindl M Di Pauli F Rostásy K Berger T. The spectrum of MOG autoantibody-associated demyelinating diseases. Nat Rev Neurol. (2013) 9:455–61. 10.1038/nrneurol.2013.118 23797245

[6] Budhram A Dubey D Sechi E Flanagan EP Yang L Bhayana V. Neural antibody testing in patients with suspected autoimmune encephalitis. Clin Chem. (2020) 66:1496–509. 10.1093/clinchem/hvaa254 33221892

[7] Dalmau J Graus F. Antibody-mediated encephalitis. N Engl J Med. (2018) 378:840–51. 10.1056/NEJMra1708712 29490181

